# Genus level molecular phylogeny of Aegisthidae Gisbrecht, 1893 (Copepoda: Harpacticoida) reveals morphological adaptations to deep-sea and plagic habitats

**DOI:** 10.1186/s12862-020-1594-x

**Published:** 2020-03-14

**Authors:** Sahar Khodami, Nancy F. Mercado-Salas, Pedro Martìnez Arbizu

**Affiliations:** Senckenberg am Meer, German Centre for Marine Biodiversity Research, Südstrand 44, 26382 Wilhelmshaven, Germany

## Abstract

**Background:**

The family Aegisthidae is known as typical component of deep-sea hyperbenthic waters that gradually colonized other marine environments. The phylogenetic relationships within this family have been examined here including hyperbenthic, planktonic, benthic forms and two associated Aegisthidae species.

**Results:**

Ninety four specimens belong to 14 genera were studied using 18S and 28S rRNA and COI mtDNA. Bayesian analysis supports the monophyly of 10 genera whereas *Andromastax, Jamstecia*, *Nudivorax* and *Aegisthus* revealed to be paraphyletic. The first offshoot of the phylogenetic tree is a clade consists of the undescribed genus Aegisthidae gen.1 sister to the two monophyletic genera *Cerviniella* and *Hase,* whereas the other Cerviniinae members (represented by *Cervinia* and *Expansicervinia*) assemble a monophylum, sister to the hyperbenthic and planktonic aegisthid genera, resulting in the paraphyly of the subfamily Cerviniinae. Hence, we defined the new subfamily Cerviniellinae subfam. nov. encompassing the three benthic genera *Cerviniella, Hase* and *Eucanuella*. The subfamily Cerviniinae has been re-defined to include *Cervinia, Expansicervinia* and *Paracerviniella*. Members of the subfamily Pontostratiotinae were clustered into two clades, one consists of the genus *Stratiopontotes* sister to an undescribed genus + *Cerviniopsis* and *Siphonis*. The second contains *Pontostratiotes* sister to the members of the planktonic subfamily Aegisthinae, resulting in the paraphyly of the Pontostratiotinae. Therefore, the Pontostratiotinae has been re-defined to include only members of the genus *Pontostratiotes*; whereas the subfamily Cerviniopseinae has been re-erected and re-defined containing *Stratiopontotes, Cerviniopsis, Siphonis*, Aegisthidae gen. 2, *Herdmaniopsis*, *Hemicervinia* and *Tonpostratiotes*. Within this subfamily, the associated *Siphonis* clusters as sister to the *Cerviniopsis* represents an example of convergent evolution in which the possession of a stylet-like mandible and an oral cone reminiscent of the Siphonostomatoida. The planktonic *Aegisthus*, *Andromastax*, *Jamstecia*, *Nudivorax* and *Scabrantenna* confirm the monophylom Aegisthinae, sister to the Pontostratiotinae.

**Conclusions:**

Our DNA based phylogeny reveals the deep-sea origin of Aegisthidae by placing benthic Aegisthidae gen.1 and Cerviniellinae subfam. nov. as the most basal lineages. Secondary adaptations to hyperbenthic and planktonic realms, as well as associated lifestyle were discovered here by the derived positions of Pontostratiotinae, Aegisthinae and *Siphonis* respectively.

## Background

It is generally believed that, ancestral copepods were living in benthic or hyperbenthic habitats [1]. The pelagic realm was colonized later during the evolution of copepods by developing specialized A1 and thoracic appendages [2–4], in association with feeding and mating behavior [5–7]. The Harpacticoida Sars G.O., 1903 is a highly successful group of Copepoda Milne Edwards, 1840 in terms of speciation and adaptations to deep sea environments [8]. Within harpacticoids, the family Aegisthidae Giesbrecht, 1893 is often the dominant taxon of deep-sea hyperbenthic communities [9–15]. Giesbrecht (1892) established the family containing two species of the mesopelagic genus *Aegisthus* Giesbrecht, 1891 *A. mucronatus* Giesbrecht, 1891 and *A. aculeatus* Giesbrecht, 1891. Later Sars (1903) established the family Cerviniidae Sars, 1903 for some benthic and hyperbenthic species. Lang [16] proposed the taxon Cerviniidimorpha Lang, 1944 to unite the Cerviniidae and the Aegisthidae into a monophyletic taxon. Several studies have mentioned taxonomic problems within the family Aegisthidae (e.g. [17–21]) but the phylogenetic relationships among the genera remained uncertain. Seifried and Schminke [22] synonymized Cerviniidae with Aegisthidae and proposed a taxonomic system of the Aegisthidae divided into three subfamilies Aegisthinae Giesbrecht, 1893, Cerviniinae Sars M., 1903 and Cerviniopseinae Brotskaya, 1963. Later Huys [23] has synonymized the Cerviniopseinae with Pontostratiotinae Scott, A., 1909 based on the principle of the priority of names, however this new arrangement was not followed by many authors and this subfamily was commonly referred as Cerviniopseinae.

In a morphology-based phylogenetic study conducted by Seifried and Schminke [22], the families Aegisthidae and Rometidae Seifried & Schminke, 2003 (= Aegisthoidea Giesbrecht, 1892) were considered as the most basal lineages of Oligoarthra Lang, 1944; however the evolutionary history within the family Aegisthidae was not studied.

Here, we attempt to resolve the phylogenetic relationships within this family based on a diverse collection of deep-sea aegisthid species inhabiting different habitats of the Atlantic and the Pacific oceans. Our molecular phylogeny of the Aegisthidae is based on small (18S) and large subunits (28S) of nuclear ribosomal RNA and the mitochondrial gene Cytochrome *c* oxidase subunit I (COI). Sixty species representing 14 genera of the three subfamilies were chosen for our phylogenetic analyses. This study represents the first attempt to reconstruct the evolutionary history of this diverse family using molecular methods and includes numerous taxa, some of them phylogenetically important, in all currently recognized subfamilies. A brief discussion of the main morphological adaptations is provided.

## Results

**Fig. 1:**
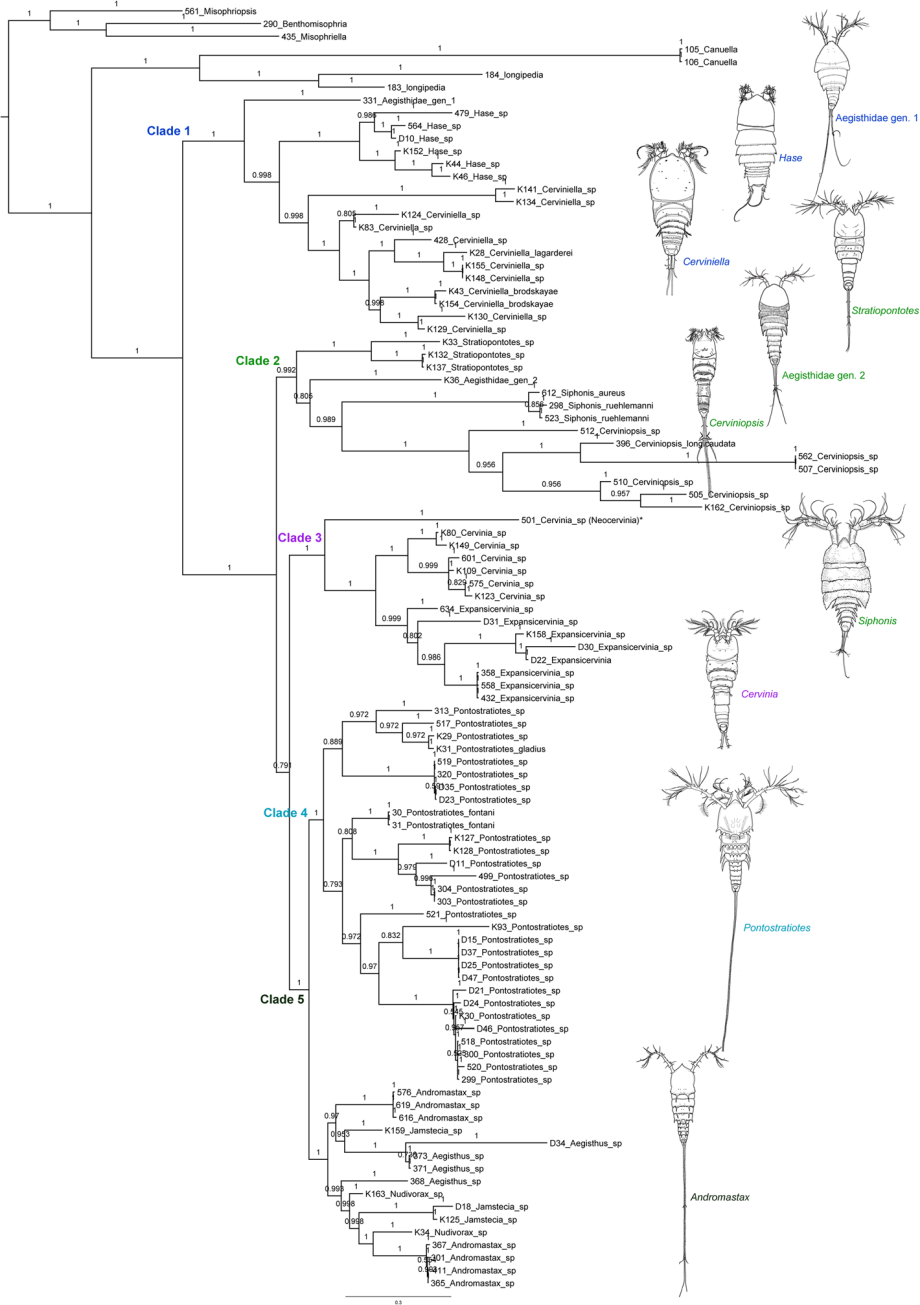
Phylogenetic relationship of 94 specimens representing 14 genera of Aegisthidae based on Bayesian analysis of 18S, 28S rRNA and COI mtDNA. The five distinct clades are marked in the tree. Nodal supports are indicated by posterior probabilities. Scale bar shows nucleotide changes per site

Bayesian phylogenetic inference using two nuclear (18S and 28S rRNA) and one mitochondrial (COI mtDNA) gene sequences recovered Aegisthidae as a monophyletic clade. Ten monophyletic genera were recovered with high support values (posterior probabilities): Aegisthidae gen. 1, *Cerviniella* Smirnov, 1946*, Hase* Corgosinho, Kihara, Schizas, Ostmann, Martinez Arbizu & Ivanenko, 2018, *Cervinia* Norman in Brady, 1878*, Expansicervinia* Montagna, 1981*, Stratiopontotes* Soyer, 1970*, Siphonis* Mercado-Salas, Khodami & Martínez Arbizu, 2019, *Cerviniopsis* Sars G.O., 1903, Aegisthidae gen. 2, and *Pontostratiotes* Brady, 1883. Our phylogeny did not support the monophyly of four genera, *Andromastax* Conroy-Dalton & Huys, 1999; *Jamstecia* Lee W. & Huys, 2000*, Aegisthus* Giesbrecht, 1891 and *Nudivorax* Lee W. & Huys, 2000*.* Among the three subfamilies previously assigned to the Aegisthidae [21, 22] only the Aegisthinae was recovered monophyletic, whereas, Cerviniinae and Pontostratiotinae were paraphyletic. The description of the new genus and species will be presented elsewhere. Figure 1 illustrates the phylogenetic tree of aegisthid species analyzed in this study in which five distinct clades have been revealed using Bayesian analysis:

### Clade 1

**Fig. 2:**
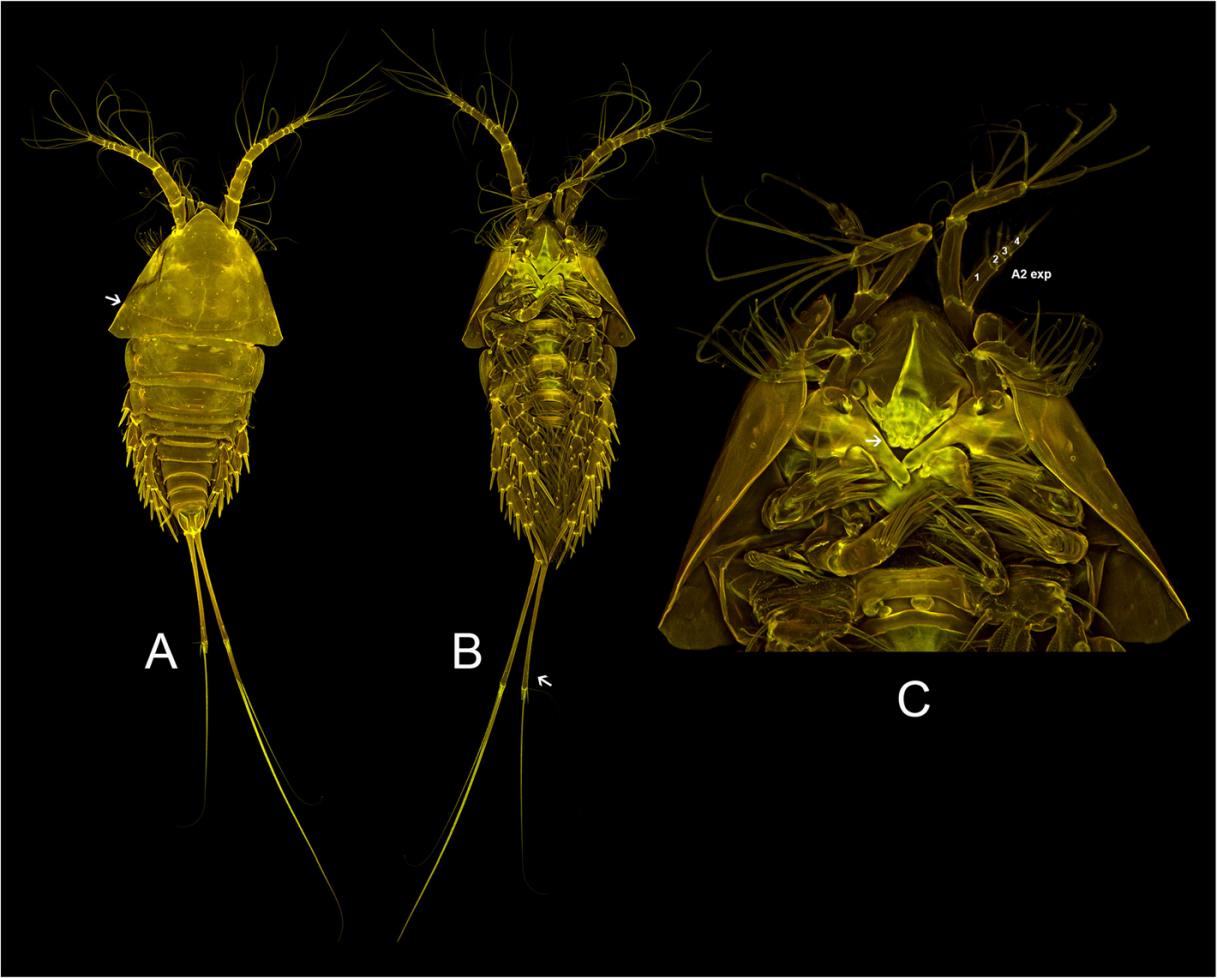
Confocal laser scanning of the Aegisthidae gen. 1, habitus, dorsal (**a**), ventral (**b**) and mouthparts (**c**)

**Fig. 3:**
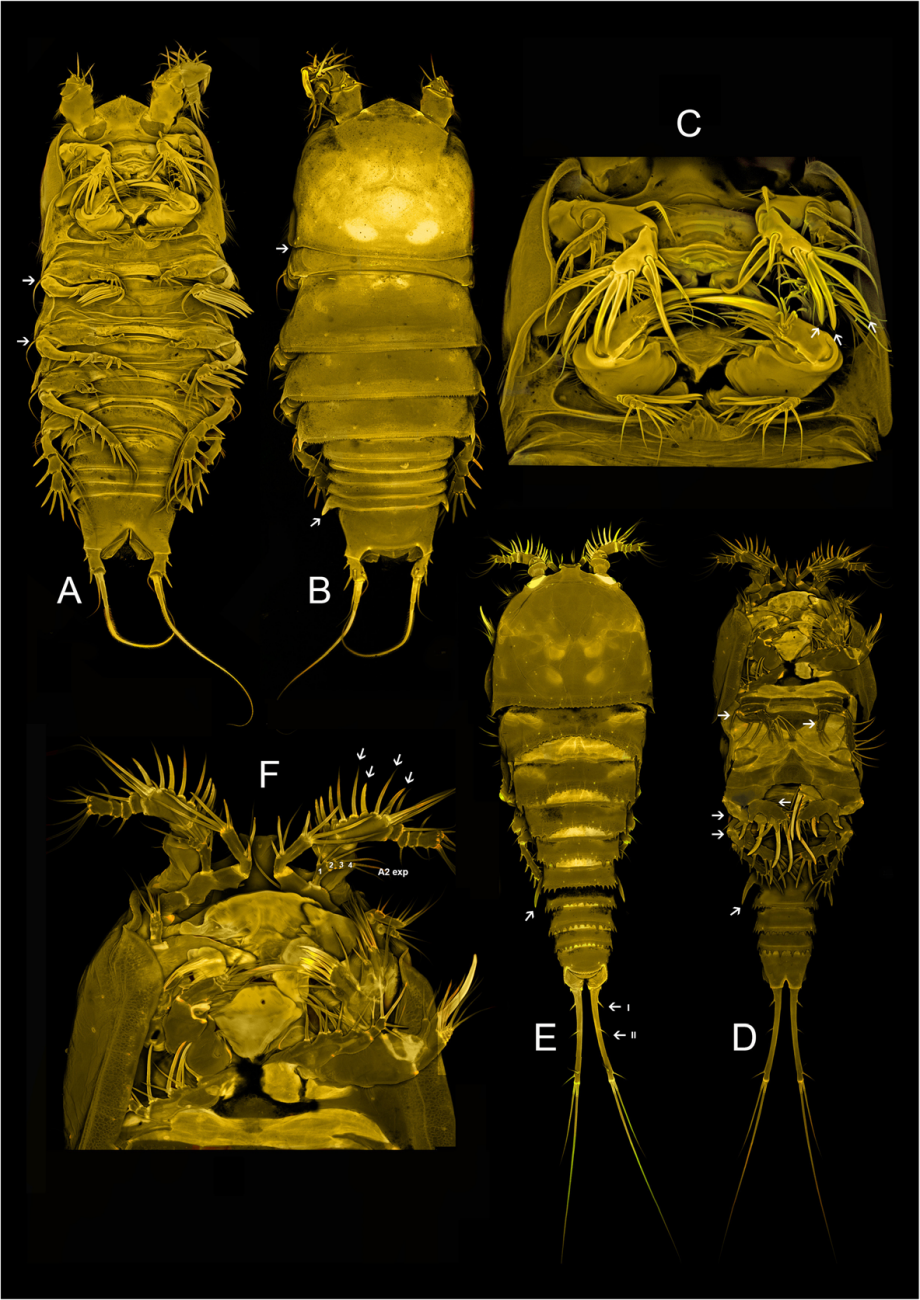
Confocal laser scanning of the taxa representing subfamily Cerviniellinae subfam. nov. *Hase talpamorphicus* Corgosinho, Kihara, Schizas, Ostmann, Martinez Arbizu & Ivanenko, 2018. habitus, dorsal (**a**), ventral (**b**) and mouthparts (**c**). *Cerviniella* sp. habitus, dorsal (**d**), ventral (**e**) and mouthparts (**f**). Images of *Hase talpamorphicus* are adapted from Corgosinho P.H.C et al. [24]

Encloses the undescribed genus Aegisthidae gen. 1 sister to a monophylum containing the benthic *Cerviniella* and *Hase*. We proposed the new subfamily Cerviniellinae subfam. nov. enclosing the genera *Cerviniella* and *Hase* (included in the molecular analysis) as well as *Eucanuella* Scott T., 1901 (based on morphological apomorphies from previous studies). The undescribed genus (Aegisthidae gen. 1) has not been included in this subfamily following distinct morphological differences, therefore retained as a single independent lineage within the clade 1. Figures 2 and 3 show confocal scanning images from habitus and mouthparts of the undescribed genus Aegisthidae gen. 1 and representatives of the subfamily Cerviniellinae (*Cerviniella* and *Hase*) respectively.

### Clade 2

**Fig. 4:**
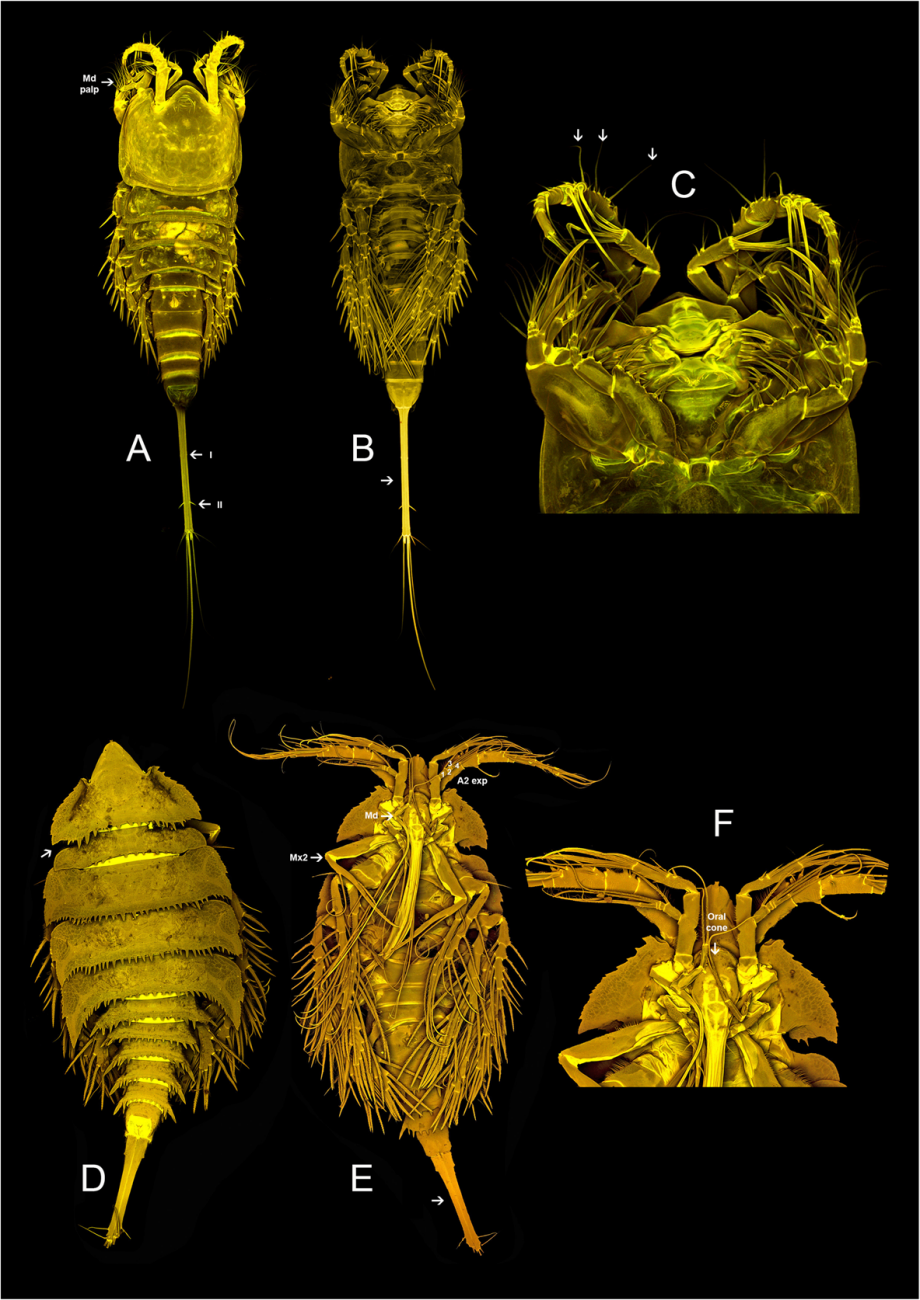
Confocal laser scanning of representatives of the subfamily Cerviniopseinae. Stratiopontotes sp. habitus, dorsal (**a**), ventral (**b**) and mouthparts (**c**). Siphonis ruehlemanni. habitus, dorsal (**d**), ventral (**e**) and mouthparts (**f**)

Contains four genera Aegisthidae gen. 2, *Cerviniopsis*, *Siphonis* and the genus *Stratiopontotes* in which the last genus was recovered sister to all remaining genera within this clade. The subfamily Cerviniopseinae has been re-erected here to contain these four genera. Also *Hemicervinia* Lang, 1935, *Herdmaniopsis* Brotskaya, 1963 and *Tonpostratiotes* Itô, 1982 are recognized here as members of the re-erected subfamily Cerviniopseinae only based on morphological evidence. The associated genus *Siphonis* has shown to be a derived lineage of Cerviniopseinae sister to *Cerviniopsis*. Figure 4 presents scanned images from the genera *Stratiopontotes* and *Siphonis.*

### Clade 3

**Fig. 5:**
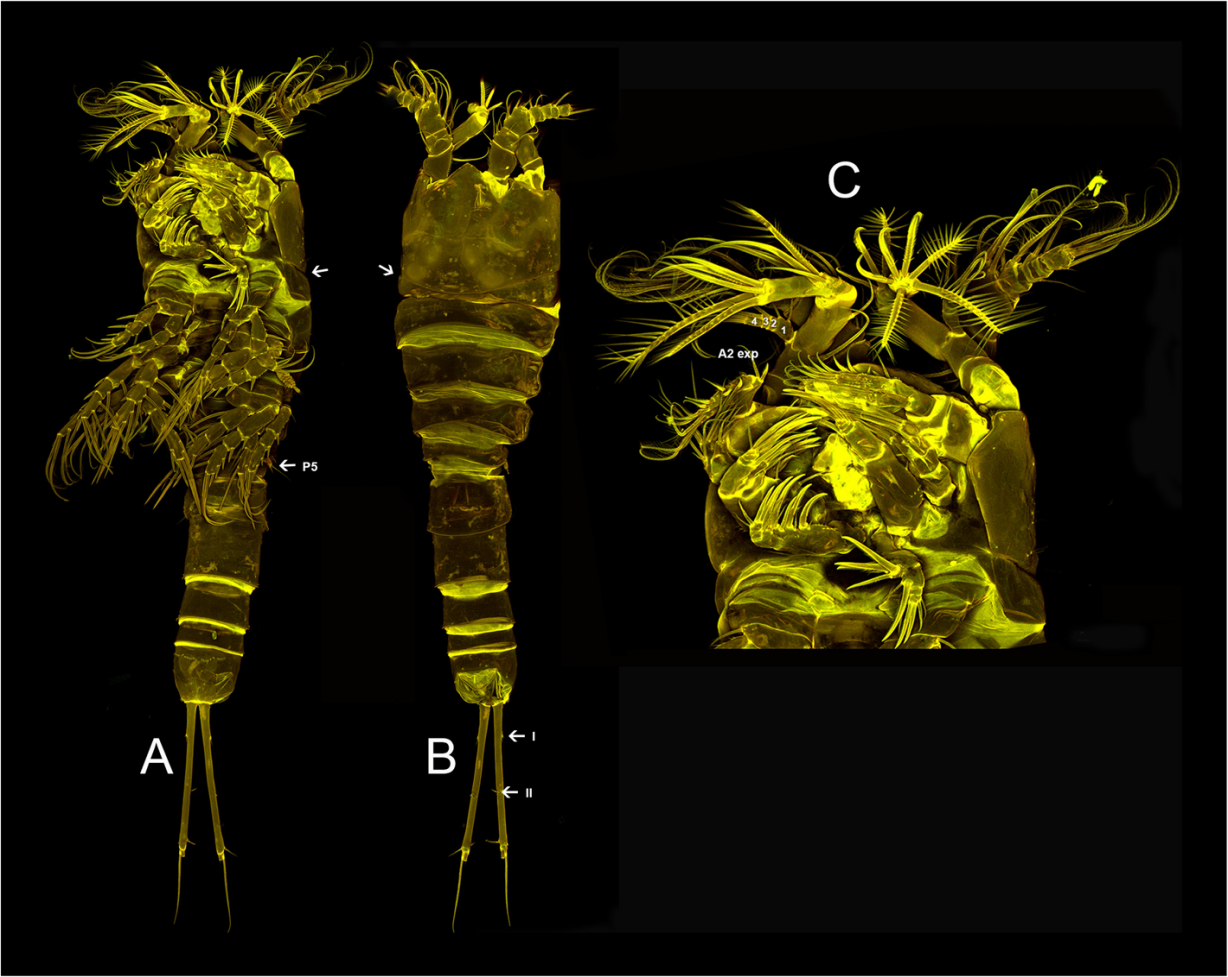
Confocal laser scanning of the specimen representing subfamily Cerviniinae. Cervinia sp. habitus, dorsal (**a**), ventral (**b**) and mouthparts (**c**)

A well-supported clade consists of two genera: *Cervinia* and *Expansicervinia.* Two distinct lineages were recovered within the genus *Cervinia*; one corresponds to the lineage previously known as *Neocervinia* Huys, Møbjerg & Kristensen, 1997(synonymized with *Cervinia* by [21]) sister to the rest of the *Cervinia.* However the both lineages are treated here as *Cervinia* due to a single specimen of the “*Neocervinia*-lineage” was available for this study. The re-validation of this taxon will be made elsewhere when enough material is available to support its taxonomic status. Here, the subfamily Cerviniinae is re-defined to enclose the genera *Cervinia, Expansicervinia* and *Paracerviniella* Brodsky, 1963 (the latest only based on morphological criteria). Figure 5 depicts the confocal scanning images form the representative of the subfamily Cerviniinae.

### Clade 4

**Fig. 6:**
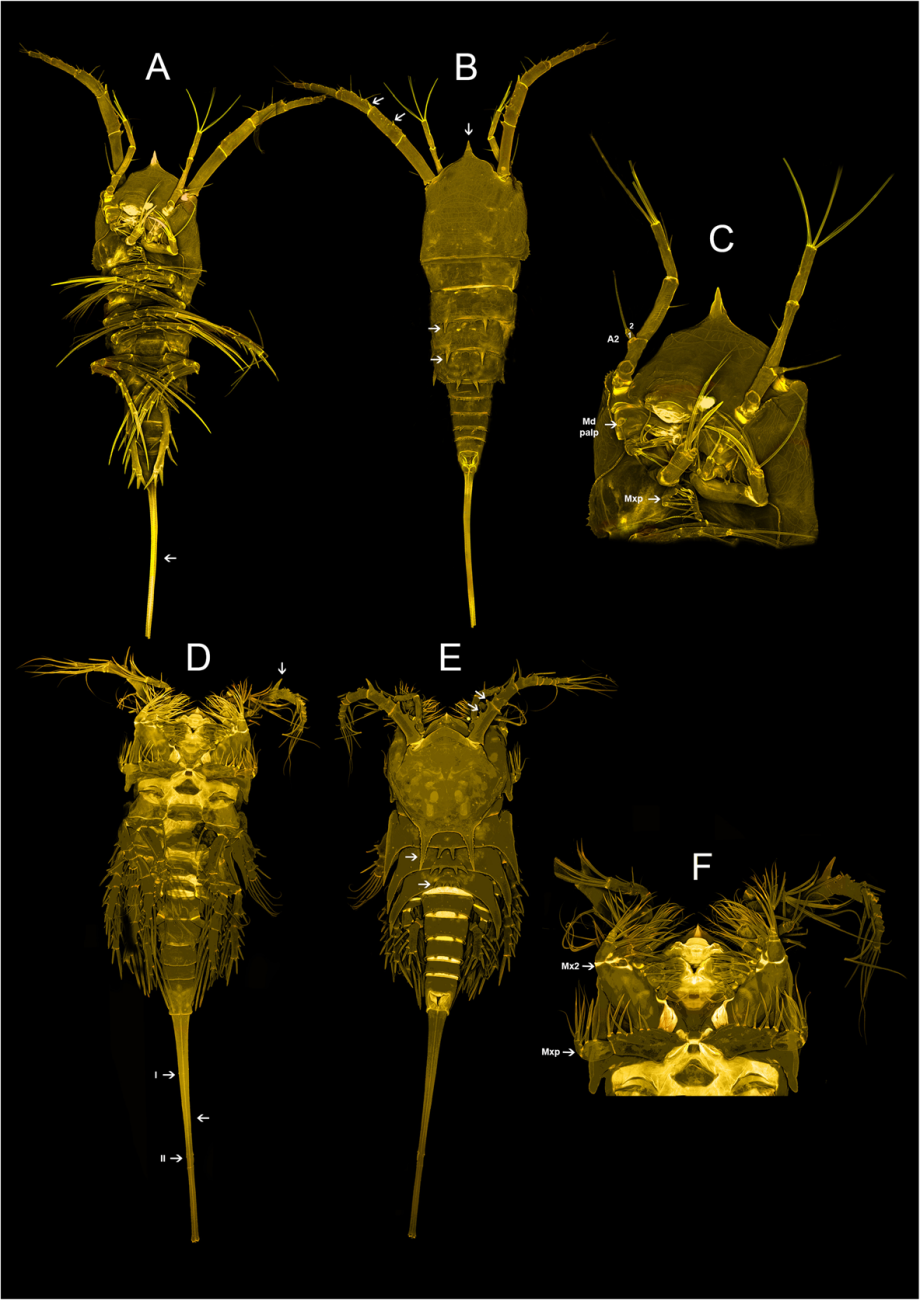
Confocal laser scanning of the species representing subfamilies Pontostratiotinae and Aegisthinae. *Jamstecia* sp. habitus, dorsal (A), ventral (B) and mouthparts (C). *Pontostratiotes* sp. habitus, dorsal (D), ventral (E) and mouthparts (F)

A highly supported monophyletic group including only members of the genus *Pontostratiotes*. Several lineages of *Pontostratiotes* were recovered which indicates the high diversity within the genus. Here we re-define the subfamily Pontostratiotinae as a monotypic taxon within Aegisthidae to accommodate species belonging to the genus *Pontostratiotes* only (Fig. 6d, e and f).

### Clade 5

Contains the genera traditionally assigned to the subfamily Aegisthinae: *Aegisthus, Andromastax, Jamstecia*, *Nudivorax* and *Scabrantenna* Lee W. & Huys, 2000 (Fig. 6a, b and c). Although this clade is highly supported to be monophyletic, the four genera, as currently defined morphologically, (*Andromastax, Aegisthus, Nudivorax* and*, Jamstecia*) are paraphyletic. *Scabrantenna* is not available in this study.

Two offbeat tree topologies have been resulted here based on two different character (nucleotide) partition settings implemented in Bayesian phylogenetic analysis from two different independent runs (see methods). The second tree topology (Additional file 1: Figure S1) contrasts with the first tree in 1) the position of Aegisthidae gen. 1, being sister to the genus *Hase,* 2) *Cerviniella* is paraphyletic, *Hase* and the Aegisthidae gen. 1 are nested within *Cerviniella* 2) the subfamilies Cerviniinae clustered as sister to an unstable clade containing Cerviniopseinae sister to Pontostratiotinae + Aegisthinae, 3) the genus *Stratiopontotes* is sister to a polytomy of *Siphonis* + Aegisthidae gen. 2 + *Cerviniopsis*, 4) the topology of the inter-clades within the genus *Pontostratiotes* is different in some lineages. This alternative phylogram displayed extremely low EES (estimated sampling size) value for the Log-likelihood estimation (LnL), it shows significantly low supports for some phylogenetically important clades and is incongruent with the principle of parsimony as accepting this topology would imply the character reversal of the unique modification of swimming legs present in *Cerviniella* (arrow in Fig. 3d) but also of the apomorphies of Cerviniellinae subfam. nov. as discussed below, to a morphologically unmodified condition in Aegisthidae gen 1. (Fig. 2b); hence this topology is presented in the supplementary material but not considered here for further discussion. The information about both MrBayes jobs including the alignments, nexus blocks, generated trees and the MrBayes log files (information about the runs) are available as Job1 and Job2 supplementary information (Additional files 2 and 3).

## Discussions

The monophyly of Aegisthidae was examined here for the first time using molecular data of 18S, 28S rRNA and COI mtDNA. Our result confirms the monophyly of the family Aegisthidae established by Seifried [21] and Seifried and Schminke [22] based on morphological characters. The traditional morphological view that, consider three monophyletic subfamilies within Aegisthidae [21, 22, 29], is not supported in the present analyses of the family (Fig. 1). Our result agrees with the hypotheses of Seifried and Schminke [22] which proposed that, the genera *Aegisthus, Andromastax, Jamstecia, Nudivorax* and *Scabrantenna* Lee W. & Huys, 2000 represent an advance possibly monophyletic group within Aegisthidae. The morphological characters that allowed the inclusion of the Aegisthinae together with former Cerviniinae and former Pontostratiotinae (sensu [30]) in the family Aegisthidae are the unique form of the anal somite (elongate and tapering posteriorly) and the modification of the posterior seta 11 (terminology following [21]) of the maxilla into a strong large spine [21, 22]. The unique spinous processes of the cephalothorax (arrow in Fig. 6b) and the extremely elongated furca (arrow in Fig. 6a) in Aegisthinae have been recognized by Seifried and Schminke [22] as derived characters shared with members of the former Pontostratiotinae (*Pontostratiotes*) (arrow in Fig. 6d,e) concluding that they belong to the same evolutionary lineage. However other harpacticoid reference literature (e.g. [29]) and even Seifried and Schminke [22] continued to consider former Pontostratiotinae and Aegisthinae to be valid, independent subfamilies. Our molecular analysis confirms the sister relationship between Aegisthinae and here re-defined Pontostratiotinae (which includes members of the *Pontostratiotes* only) and rejects the monophyly of the former Pontostratiotinae.

In this study, the analyzed genera belonging to the Aegisthinae (*Aegisthus, Andromastax, Nudivorax,* and *Jamstecia*) have a complex topology (Fig. 1) in which there is no evidence supporting the monophyly of none of the four genera. Conroy Dalton and Huys [27] described *Andromastax* as the second known genus in the Aegisthidae, arguing that the inclusion of the new species within *Aegisthus* (the only genus recognized at that time) was impossible without grossly extending its generic boundaries. Some of the morphological characters used to define *Andromastax* were based on a combination of strong plesiomorphic character states (e.g. Md palp 2-segmented and bisetose; Mx1 basis with 8 elements; A2 exopod with 3 elements; P5 exopod with inner setae; P6 with 3 setae) and clearly defined apomorphies (e.g. dorsal spinous processes on cephalothorax, cephalosome with lateral spinous processes near bases of A2; lateral processes on coxae P2-P4, ♀ second segment of A1 with 2 lateral processes on anterior margin, ♂ Mx2 allobasis and enp-1 with modified pinnate spines [27]. We suggest that the dorsal spinous processes on the trunk somites and A1 (arrow in Fig. 6b, e) represent a synapomorphic character for the lineage formed by Aegisthinae + Pontostratiotinae which were lost in some members of *Pontostratiotes* and in the genus *Nudivorax.* Lee and Huys [19] described three monotypic genera of Aegisthinae from hydrothermal vents and cold seeps in Japan: *Nudivorax, Scabrantenna* and *Jamstecia*. *Nudivorax* is characterized by a complete lack of integumental surface reticulation and lack of spinous processes on both cephalosome and body somites which was considered to be unique among the Aegisthinae. *Scabrantenna* (missing from this analysis) is distinguished from other genera because of its sexually dimorphic A1 and its prehensile A2 (presumably used for mate guarding). The most distinctive character of *Jamstecia* is an elongated A1, resulting from secondary elongation of segment 1 (Fig. 6a, b), differing from other Aegisthinae in which the second segment is the longest. Lee and Huys [19] argued that *Nudivorax* could represent the most primitive genus within the family because of the plesiomorphic state of female A1 (which retains the maximum number of setae expressed in the family) and male mouthparts (retaining the full complement of armature as found in females) suggesting an early divergence within the subfamily. The above mentioned authors also discussed that *Scabrantenna* represents a transitional genus between the primitive *Nudivorax* and *Andromastax* compared with the advanced *Aegisthus* and questioned the position of the genus *Jamstecia* due to the lack of material for comparison. Lack of support in the monophyly of these four genera in our study demands providing DNA sequences from the type species of these genera for deeper molecular and morphological analysis. The materials we have sequenced correspond morphologically to the genera assigned in Fig. 1 but the species are not the name bearing types.

Here we, re-validate and re-define the subfamily Cerviniopseinae as a well-supported monophyletic group that includes the genera *Stratiopontotes, Siphonis*, *Cerviniopsis,* Aegisthidae gen. 2., *Hemicervinia*, *Herdmaniopsis* and *Tonpostratiotes*. *Stratiopontotes* is here shown to be the most basal genus within the subfamily Cerviniopseinae. This genus mostly follows the ground pattern of the Aegisthidae in both cephalic and thoracic appendages, converging the molecular analysis with the morphology. Members of the genus *Herdmaniopsis* have been recognized as primitive forms within the former Pontostratiotinae by Lang [31], Brotskaya [9] and later by Ito [28]. The last author particularly emphasized the morphological similarities of the unarmed cephalic shield and thoracic pleuro-tergites of *Stratiopontotes* (Fig. 4a) and *Herdmaniopsis*, suggesting the close relationship between those genera. *Herdmaniopsis* has no representative in our molecular study, however we agree with Ito [28] and believe that *Herdmaniopsis* may be sister to *Stratiopontotes* in a basal position within Cerviniopseinae. Additionally, we agree with the clear separation of both genera because of the absence of setae on the first endopodite of the maxilliped and the shortened of the A1 in *Herdmaniopsis,* among other characters.

The only known associated genus of the family Aegisthidae, *Siphonis,* is shown here to be sister to *Cerviniopsis*. This genus is characterized by its siphonostomatoid-like mouthparts which include an elongated oral cone (formed by labrum and labium; that are sealed together by complex arrangement of overlapping ridges and grooves), a highly modified mandible (stylet-like) and maxillule and maxilla with the same shape as in siphonostomatoids (arrow in Fig. 4e,f) [26]. Those analogous structures have previously been considered synapomorphies for the taxon Palinarthra Seifried, 2003 within the Harpacticoida [21]. The Palinarthra includes the families Novocriniidae Huys & Iliffe, 1998, Superornatiremidae Huys, 1996, Rotundiclipeidae Huys, 1988, Peltidiidae Claus, 1860, Tegastidae Sars, G. O. 1904, and Porcellidiidae Boeck, 1865 as well as the families within the superfamily Tisboidea Stebbing, 1910 [21]. The short oral cone in Paninarthra families shows a similar morphology to the primitive families Asterocheridae Giesbrecht, 1899 and Dirivultidae Humes and Dojiri, 1980 of Siphonostomatoida [32, 33]; whereas, an elongated oral cone and highly modified mandible in *Siphonis* is considered a homoplasy with some particular Asterocheridae species such as *Acontiophorus scutatus* (Brady and Robertson, 1873) and *Scottocheres elongatus* (Scott T. & Scott A., 1894) both associated with Porifera. These similar modifications are characteristic of those associated copepods and have the functional task of penetrating and feeding from pores and channels in sponges (for a detailed discussion of the morphology and mouthpart modifications in the genus *Siphonis* see [26]).

Aegisthidae gen. 1 is, genetically, a well-supported independent lineage, sister to the subfamily Cerviniellinae subfam. nov. This undescribed genus was excluded from the Cerviniellinae subfam. nov. based on the distinct morphological differences between this genus compare to *Hase* and *Cerviniella*. The taxonomic status of the undescribed genus needs to be studied in details when additional material become available. Furthermore this genus is morphologically similar to the genus *Stratiopontotes* in the general armature and segmentation of A1, A2, mandible, maxillula, maxilla, maxilliped and P1-P5 (Fig. 2b, c). However it can be easily distinguished from *Stratiopontotes* because of its divergent furca (arrow in Fig. 2b), the robust and slightly projected labrum (arrow in Fig. 2c), the ratio of P1 endopod-exopod and; its general shape -being more robust and moderately dorso-ventrally flattened than *Stratiopontotes-* among other characters. Morphologically, the undescribed genus Aegisthidae gen. 1 agrees with the ground pattern of Harpacticoida in several important characters; one of them is the incomplete fusion of the first pedigerous somite from the dorsal cephalic shield (arrow in Fig. 2a shows dorsal suture). The complete fusion of the above mentioned somites has been considered as synapomorphy for the Syngnatharthra group which includes all families within Harpacticoida except for Aegisthidae and Rometidae [22]. However a complete secondary separation of the cephalosome and first pedigerous somite is presented in some species of Syngnatharthra as Chappuisiidae Chappuis, 1940, Phyllognathopodidae Gurney, 1932 and Latiremidae Bozic, 1969; or partially retained in members of Rotundiclipeidae and Tachidiidae Sars, G. O. 1909.

The subfamily Cerviniellinae subfam. nov. is here re-defined to enclose members of the genus *Cerviniella, Hase* and *Eucanuella*, the latest is not included in the molecular analysis. *Eucanuella* is included within this subfamily because of the fusion of the first pedigerous somite to the cephalosome (similar to *Cerviniella*; arrow in Fig. 3e), the short A1 bearing several elements transformed into spines (arrow in Fig. 3f), the presence of strong hook-like lateral projections on the genital double somite (arrow in Fig. 3e,f) and the P1 exp. bended inwards (similar to *Hase*; arrow in Fig. 3a), the last three characters considered here as apomorphies to the Cerviniellinae subfam. Nov.

The genera *Neocervinia* and *Pseudocervinia* were synonymized with *Cervinia*, based on a ‘strict consensus tree’ [21], who considered the female one segmented P5 (arrow in Fig. 5a) as the only apomorphic character of *Neocervinia*. Later, Park et al. [20] endorsed the generic status of *Neocervinia* adding the presence of an aesthetasc (incorrectly referred by the authors as sensilla) on the A1 second and third segments as another synapomorphy for the genus (see [19, 34] for detailed characters). Our molecular analysis is incongruent with synonymizing *Neocervinia* with *Cervinia*, however re-validation of this taxon is postponed providing more material from this genus.

## Conclusions

**Fig. 7:**
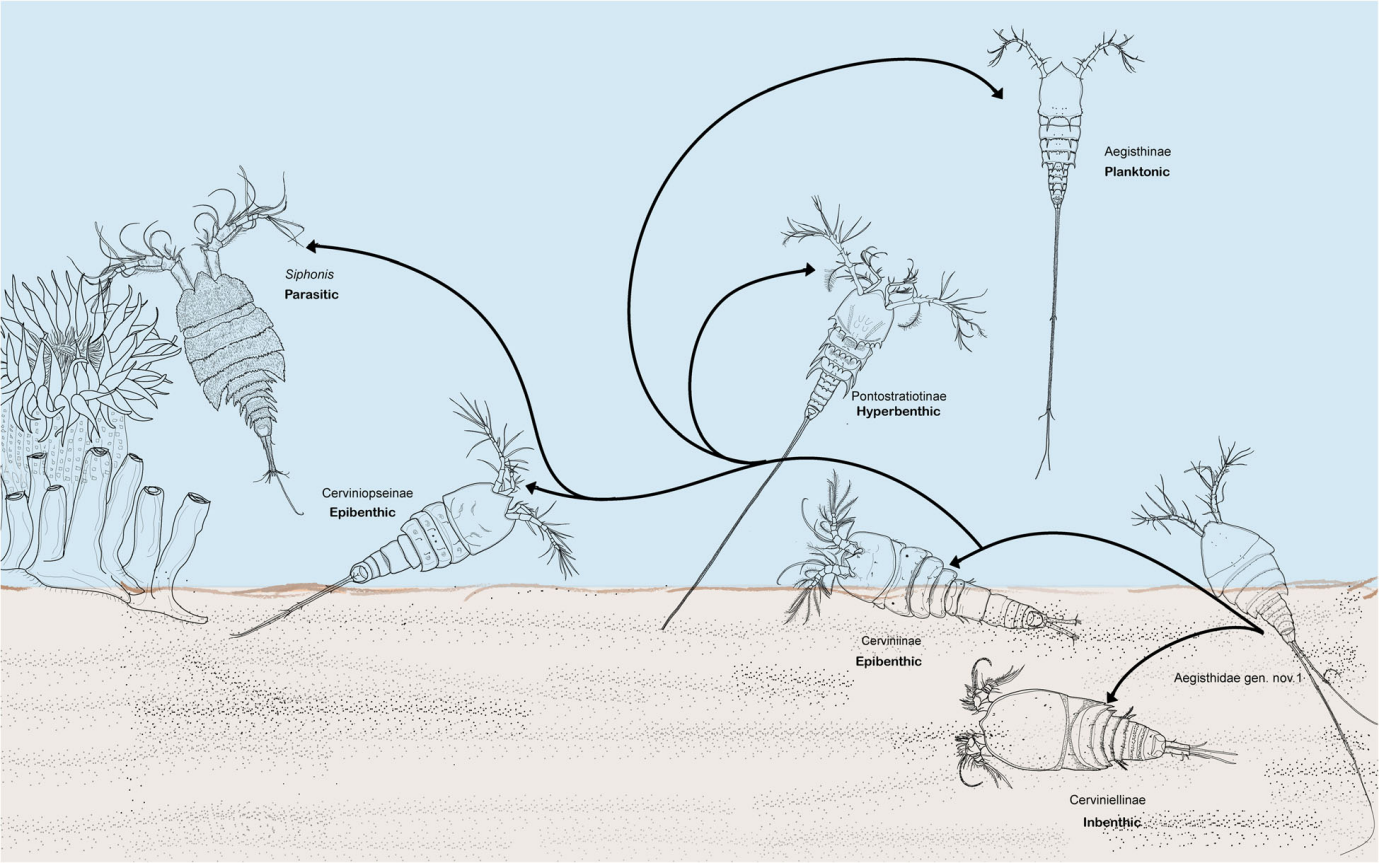
The ecological adaptations of the Aegisthidae, showing the gradually colonized habitats by line drawings of the representative morphotype of each branch. The arrows show the evolutionary history considered for the Aegisthidae from its benthic ancestors

The family Aegisthidae has colonized a variety of habitats in the course of its diversification (Fig. 7). The deep-sea benthic origin of Aegisthidae [9, 35] is demonstrated by the basal lineages such as Aegisthidae gen. 1 and Cerviniellinae subfam. nov. These species live on and in the sediments, but may also swim in the water close to the bottom (epibenthic). Broad spade-like appendages which are associated with burrowing lifestyle [13, 36, 37] are adaptations in benthic Cerviniellinae subfam. nov. species. These adaptations involve a stout and robust body shape, commonly with short furca, reduction in the length of the A1 segments (always less than one fifth of the total length of cephalosome), the development of robust modified setae in the A1, reductions in swimming legs (e.g. P1 endopod reduced to a single segment or absent in some species), swimming legs bent inwardly and with strongly developed spines and processes that may help in sediment removal.

Secondary adaptations to a pelagic existence in Aegisthidae [25, 38] are clearly distinguished in Pontostratiotinae in which extremely long furca, well developed spinous process and an increased body surface aid in avoidance of sinking are present. Members of the genus *Pontostratiotes* inhabit hyperbenthic water layers. The derived Aegisthinae, such as the planktonic species of the genus *Aegisthus* the increase in the length of the A1, A2 and furca and the segments of swimming legs are shown here to play an important role in the successful colonization of pelagic realm of the ocean [8, 9, 13, 25, 31, 38]. The most advanced life style in this family is displayed by *Siphonis*, where mouth parts have been adapted to an associated life style, with a long siphonostomatoid-like oral cone, stylet-like mandible and siphostomatoid-like maxillula. Here, a comprehensive molecular phylogeny of the Aegisthidae shows that evolutionary trends in this family have begun from a benthic habit then to an epibenthic, hyperbenthic, planktonic then associated lifestyle.

## Methods

### Taxon sampling

The Aegisthidae species analyzed for this study were collected during four research cruises. The Atlantic Ocean were sampled around Iceland during the cruise ME 85–3 IceAGE onboard *RV Meteor* ([39] https://www.ldf.uni-hamburg.de/meteor/wochenberichte.html). Most specimens included in this study were obtained from three other cruises in the Pacific Ocean by *RV SONNE*, the first in the abyssal and hadal zones of the Kuril-Kamchatka Trench SO250 ([40] 10.4126/FRL01-006401131), the second in the Clarion Clipperton Fracture Zone, SO239 ([41] http://oceanrep.geomar.de/30422/) and the third, SO242–1 ecological aspect of deep-sea mining in the manganese nodule area of the south east Pacific ([42]; https://www.portal-forschungsschiffe.de/lw_resource/datapool/_items/item_148/so-242_1_fahrtbericht.pdf.)

Samples were preserved in 96% ethanol and specimens were sorted using a dissecting microscope. Aegisthidae specimens were isolated and stored in 96% ethanol at − 20 °C. Species were identified to the lowest taxonomic level using diagnostic morphological characteristics. Many of the collected species are new to science and not yet described. Ninety four specimens were available representing the currently valid subfamilies of Aegisthidae: Aegisthinae (10 species) comprising 4 genera, *Aegisthus, Andromastax, Jamstecia* and *Nudivorax*; Cerviniinae (18 species) including 6 genera, Aegisthidae gen. 1, *Cerviniella, Neocervinia*, *Cervinia, Expansicervinia* and *Hase*; Pontostratiotinae (20 species) including 5 genera, *Stratiopontotes*, *Siphonis*, *Cerviniopsis, Pontostratiotes* and Aegisthidae gen. 2. Table 1 shows collected taxa and sampling coordinates.

**Table 1:** Taxon sampling of the Aegisthidae species collected and sequenced for this study. Sampling stations and coordinates are listed for each specimen

ID	Taxon	Expedition	Location	Station	Latitude	Longitude	Depth (m)
331	Aegisthidae gen.1	SO239	Clarion Clipperton Fracture Zone	21	11° 51,21′ N	117° 3,57′ W	4146
K43	*Cerviniella brodskayae*	SO250	Kuril-Kamchatka Trench	25	45° 55,237′ N	152° 47,467′ E	6066.4
K154	*Cerviniella brodskayae*	SO250	Kuril-Kamchatka Trench	10	43° 51,810′ N	151° 46,543′ E	5188.1
K129	*Cerviniella* sp.	SO250	Kuril-Kamchatka Trench	61	45° 9997′ N	153° 45,417′ E	5740,8 0,3
K130	*Cerviniella* sp.	SO250	Kuril-Kamchatka Trench	61	45° 9997′ N	153° 45,417′ E	5740,8 0,3
428	*Cerviniella* sp.	SO239	Clarion Clipperton Fracture Zone	81	11° 3,97′ N	119° 37,67′ W	4365.7
K28	*Cerviniella lagarderei*	SO250	Kuril-Kamchatka Trench	8	43° 51,698′ N	151° 45,851′ E	5191,2 0,3
K148	*Cerviniella* sp.	SO250	Kuril-Kamchatka Trench	10	43° 51,810′ N	151° 46,543′ E	5188.1
K155	*Cerviniella* sp.	SO250	Kuril-Kamchatka Trench	10	43° 51,810′ N	151° 46,543′ E	5188.1
K83	*Cerviniella* sp.	SO250	Kuril-Kamchatka Trench	17	45° 54,160′ N	153° 54,685’	7994
K124	*Cerviniella* sp.	SO250	Kuril-Kamchatka Trench	42	45° 37,602′ N	152° 52,499′ E	6881.4
K134	*Cerviniella* sp.	SO250	Kuril-Kamchatka Trench	62	45° 9998′ N	153° 45,418′ E	5742.5
K141	*Cerviniella* sp.	SO250	Kuril-Kamchatka Trench	53	45° 28,751′ N	153° 11,649′ E	8941.4
564	*Hase* sp.	SO239	Clarion Clipperton Fracture Zone	124	13° 51,28′ N	123° 14,69′ W	4510.8
D10	*Hase* sp.	SO242–1	Manganese Nodule area of the South East Pacific	45	7° 07,116′ S	88° 26,356′ W	4184
479	*Hase* sp.	SO239	Clarion Clipperton Fracture Zone	50	11° 49,92′ N	117° 29,31′ W	4330.3
K152	*Hase* sp.	SO250	Kuril-Kamchatka Trench	10	43° 51,810′ N	151° 46,543′ E	5188.1
K46	*Hase* sp.	SO250	Kuril-Kamchatka Trench	10	43° 51,810′ N	151° 46,543′ E	5188.1
K44	*Hase* sp.	SO250	Kuril-Kamchatka Trench	8	43° 51,698′ N	151° 45,851′ E	5191,2 0,3
501	*Neocervinia* sp.	SO239	Clarion Clipperton Fracture Zone	99	11° 2,28′ N	119° 40,89′ W	4401.4
358	*Expansicervinia* sp.	SO239	Clarion Clipperton Fracture Zone	20	11° 50,31′ N	116° 58,78′ W	4093
558	*Expansicervinia* sp.	SO239	Clarion Clipperton Fracture Zone	94	11° 4,42′ N	119° 39,33′ W	4414.4
432	*Expansicervinia* sp.	SO239	Clarion Clipperton Fracture Zone	81	11° 4,29′ N	119° 36,29′ W	4346.4
D31	*Expansicervinia* sp.	SO242–1	Manganese Nodule area of the South East Pacific	37	7° 07,854′ S	88° 25,484′ W	4176.3
634	*Expansicervinia* sp.	SO239	Clarion Clipperton Fracture Zone	117	13° 52,78′ N	123° 13,82′ W	4513.1
K80	*Cervinia* sp.	SO250	Kuril-Kamchatka Trench	17	45° 54,160′ N	153° 54,685’	7994
K149	*Cervinia* sp.	SO250	Kuril-Kamchatka Trench	52	45° 31,996′ N	153° 15,993′ E	8358.4
601	*Cervinia* sp.	SO239	Clarion Clipperton Fracture Zone	158	14° 3,41′ N	130° 7,99′ W	4946
575	*Cervinia* sp.	SO239	Clarion Clipperton Fracture Zone	118	13° 52,38′ N	123° 15,09′ W	4511.7
K123	*Cervinia* sp.	SO250	Kuril-Kamchatka Trench	42	45° 37,602′ N	152° 52,499′ E	6881.4
K109	*Cervinia* sp.	SO250	Kuril-Kamchatka Trench	40	45° 39,976′ N	152° 55,953′ E	7300.3
D22	*Expansicervinia* sp.	SO242–1	Manganese Nodule area of the South East Pacific	93	6° 59,902′ S	88° 30;764′ W	4142.1
K158	*Expansicervinia* sp.	SO250	Kuril-Kamchatka Trench	10	43° 51,810′ N	151° 46,543′ E	5188.1
D30	*Expansicervinia* sp.	SO242–1	Manganese Nodule area of the South East Pacific	37	7° 07,854 S	88° 25,484′ W	4176.3
K33	*Stratiopontotes* sp.	SO250	Kuril-Kamchatka Trench	8	43° 51,698′ N	151° 45,851′ E	5191,2 0,3
K137	*Stratiopontotes* sp.	SO250	Kuril-Kamchatka Trench	62	45° 9998′ N	153° 45,418′ E	5742.5
K132	*Stratiopontotes* sp.	SO250	Kuril-Kamchatka Trench	61	45° 9997′ N	153° 45,417′ E	5740,8 0,3
523	*Siphonis ruehlemanni*	SO239	Clarion Clipperton Fracture Zone	20	11° 50,31′ N	116° 58,78′ W	4093
298	*Siphonis ruehlemanni*	SO239	Clarion Clipperton Fracture Zone	20	11° 50,31′ N	116° 58,78′ W	4093
543	*Siphonis aureus*	SO239	Clarion Clipperton Fracture Zone	81	11° 4,29′ N	119° 36,29′ W	4346.4
521	*Cerviniopsis* sp.	SO239	Clarion Clipperton Fracture Zone	20	11° 50,31′ N	116° 58,78′ W	4093
396	*Cerviniopsis longicaudata*	SO239	Clarion Clipperton Fracture Zone	59	11° 48,22′ N	117° 30,42′ W	4324.5
507	*Cerviniopsis* sp.	SO239	Clarion Clipperton Fracture Zone	99	11° 2,28′ N	119° 40,89′ W	4401.4
562	*Cerviniopsis* sp.	SO239	Clarion Clipperton Fracture Zone	99	11° 2,28′ N	119° 40,89′ W	4401.4
510	*Cerviniopsis* sp.	SO239	Clarion Clipperton Fracture Zone	99	11° 2,28′ N	119° 40,89′ W	4401.4
K196	*Cerviniopsis* sp.	SO250	Kuril-Kamchatka Trench	86	45° 1202′ N	151° 6008′ E	5571.6
505	*Cerviniopsis* sp.	SO239	Clarion Clipperton Fracture Zone	99	11° 2,28′ N	119° 40,89′ W	4401.4
K36	Aegisthidae gen.2	SO250	Kuril-Kamchatka Trench	8	43° 51,698′ N	151° 45,851′ E	5191,2 0,3
K163	*Nudivorax* sp.	SO250	Kuril-Kamchatka Trench	55	45° 29,242′ N	153° 13,453′ E	8734.4
K125	*Jamstecia* sp.	SO250	Kuril-Kamchatka Trench	5	43° 49,196′ N	151° 45,593′ E	5149.4
D18	*Jamstecia* sp.	SO242–1	Manganese Nodule area of the South East Pacific	81	7 °03,442 S	88° 28,903′ W	4152.9
301	*Andromastax* sp.	SO239	Clarion Clipperton Fracture Zone	20	11° 50,31′ N	116° 58,78′ W	4093
411	*Andromastax* sp.	SO239	Clarion Clipperton Fracture Zone	50	11° 49,92′ N	117° 29,31′ W	4330.3
365	*Andromastax* sp.	SO239	Clarion Clipperton Fracture Zone	20	11° 50,31′ N	116° 58,78′ W	4093
367	*Andromastax* sp.Andr	SO239	Clarion Clipperton Fracture Zone	20	11° 50,31′ N	116° 58,78′ W	4093
K34	*Nudivorax* sp.	SO250	Kuril-Kamchatka Trench	8	43° 51,698′ N	151° 45,851′ E	5191,2 0,3
368	*Aegisthus* sp.	SO239	Clarion Clipperton Fracture Zone	45	7° 07,116′ S	88° 26,356′ W	4184
K159	*Jamstecia* sp.	SO250	Kuril-Kamchatka Trench	10	43° 51,810′ N	151° 46,543′ E	5188.1
373	*Aegisthus* sp.	SO239	Clarion Clipperton Fracture Zone	20	11° 50,31′ N	116° 58,78′ W	4093
371	*Aegisthus* sp.	SO239	Clarion Clipperton Fracture Zone	20	11° 50,31′ N	116° 58,78′ W	4093
D34	*Aegisthus* sp.	SO242–1	Manganese Nodule area of the South East Pacific	37	7° 07,854 S	88° 25,484′ W	4176.3
619	*Andromastax* sp.	SO239	Clarion Clipperton Fracture Zone	118	13° 52,38′ N	123° 15,09′ W	4511.7
616	*Andromastax* sp.	SO239	Clarion Clipperton Fracture Zone	171	14° 2,68′ N	130° 5,97′ W	5030.2
576	*Andromastax* sp.	SO239	Clarion Clipperton Fracture Zone	118	13° 52,38′ N	123° 15,09′ W	4511.7
K128	*Pontostratiotes* sp.	SO250	Kuril-Kamchatka Trench	23	45° 57,724′ N	152° 39,836′ E	5959.2
K127	*Pontostratiotes* sp.	SO250	Kuril-Kamchatka Trench	23	45° 57,724′ N	152° 39,836′ E	5959.2
304	*Pontostratiotes* sp.	SO239	Clarion Clipperton Fracture Zone	20	11° 50,31′ N	116° 58,78′ W	4093
303	*Pontostratiotes* sp.	SO239	Clarion Clipperton Fracture Zone	20	11° 50,31′ N	116° 58,78′ W	4093
499	*Pontostratiotes* sp.	SO239	Clarion Clipperton Fracture Zone	99	11° 2,28′ N	119° 40,89′ W	4401.4
D11	*Pontostratiotes* sp.	SO242–1	Manganese Nodule area of the South East Pacific	37	7° 07,854 S	88° 25,484′ W	4176.3
30	*Pontostratiotes fontani*	ME 85–3	North Atlantic around Iceland	1054	61° 57,33 N	31°38,583′W	2548
31	*Pontostratiotes fontani*	ME 85–3	North Atlantic around Iceland	1054	61° 57,33 N	31°38,583′W	2548
521	*Pontostratiotes* sp.	SO239	Clarion Clipperton Fracture Zone	20	11° 50,31′ N	116° 58,78′ W	4093
D46	*Pontostratiotes* sp.	SO242–1	Manganese Nodule area of the South East Pacific	45	7° 07,116′ S	88° 26,356′ W	4184
520	*Pontostratiotes* sp.	SO239	Clarion Clipperton Fracture Zone	20	11° 50,31′ N	116° 58,78′ W	4093
518	*Pontostratiotes* sp.	SO239	Clarion Clipperton Fracture Zone	20	11° 50,31′ N	116° 58,78′ W	4093
300	*Pontostratiotes* sp.	SO239	Clarion Clipperton Fracture Zone	20	11° 50,31′ N	116° 58,78′ W	4093
D24	*Pontostratiotes* sp.	SO242–1	Manganese Nodule area of the South East Pacific	81	7 °03,442 S	88° 28,903′ W	4152.9
D21	*Pontostratiotes* sp.	SO242–1	Manganese Nodule area of the South East Pacific	81	7 °03,442 S	88° 28,903′ W	4152.9
K30	*Pontostratiotes* sp.	SO250	Kuril-Kamchatka Trench	8	43° 51,698′ N	151° 45,851′ E	5191,2 0,3
299	*Pontostratiotes* sp.	SO239	Clarion Clipperton Fracture Zone	20	11° 50,31′ N	116° 58,78′ W	4093
K93	*Pontostratiotes* sp.	SO250	Kuril-Kamchatka Trench	30	45° 56,838′ N	152° 50,939′ E	6165.1
K31	*Pontostratiotes gladius*	SO250	Kuril-Kamchatka Trench	8	43° 51,698′ N	151° 45,851′ E	5191,2 0,3
517	*Pontostratiotes* sp.	SO239	Clarion Clipperton Fracture Zone	20	11° 50,31′ N	116° 58,78′ W	4093
D15	*Pontostratiotes* sp.	SO242–1	Manganese Nodule area of the South East Pacific	81	7 °03,442 S	88° 28,903′ W	4152.9
D47	*Pontostratiotes* sp.	SO242–1	Manganese Nodule area of the South East Pacific	93	6° 59,902′ S	88° 30;764′ W	4142.1
D25	*Pontostratiotes* sp.	SO242–1	Manganese Nodule area of the South East Pacific	81	7 °03,442 S	88° 28,903′ W	4152.9
D37	*Pontostratiotes* sp.	SO242–1	Manganese Nodule area of the South East Pacific	45	7° 07,116′ S	88° 26,356′ W	4184
519	*Pontostratiotes* sp.	SO239	Clarion Clipperton Fracture Zone	20	11° 50,31′ N	116° 58,78′ W	4093
320	*Pontostratiotes* sp.	SO239	Clarion Clipperton Fracture Zone	24	11° 51,87′ N	116° 59,74′ W	4122
D23	*Pontostratiotes* sp.	SO242–1	Manganese Nodule area of the South East Pacific	45	7° 07,116′ S	88° 26,356′ W	4184
D35	*Pontostratiotes* sp.	SO242–1	Manganese Nodule area of the South East Pacific	45	7° 07,116′ S	88° 26,356′ W	4184
K29	*Pontostratiotes sp.*	SO250	Kuril-Kamchatka Trench	8	43° 51,698′ N	151° 45,851′ E	5191,2 0,3
313	*Pontostratiotes sp.*	SO239	Clarion Clipperton Fracture Zone	20	11° 50,31′ N	116° 58,78′ W	4093

### Molecular analysis

DNA extractions were carried out using 40 μL Chelex (InstaGene Matrix, Bio − Rad) following the protocol of Estoup et al. [43] from whole individuals and supernatant was stored at − 20 °C for later DNA analysis. Exoskeletons of the extracted specimens were fixed in glycerin on a glass slide and stored as a voucher for morphological identifications. DNA was analyzed for all 94 specimens available for this study (Table 1). Phylogenetic analyses were carried out using 914 bp of nuclear large (28S), 1792 bp of small (18S) subunits of rRNA and 741 bp of mitochondrial protein cytochrome *c* oxidase subunit I (COI). Amplification was performed using AccuStart II GelTrack PCR SuperMix (ThermoFisher Scientific) or Phire Green Hot Start II PCR Master Mix (ThermoFisher Scientific) in a 25 μL volume containing 9.5 μL H2O, 12.5 μL PCR Master Mix, 0.5 μL of each primer (10 pmol μL^− 1^) and 2 μL of DNA template. PCR products were checked by electrophoresis on a 1% agarose/TAE gel containing 1x GelRed. PCR products have been purified using EXO SAP (PCR cleaning, ThermoFisher Scientific) prior sequencing. Table 2 shows related PCR primers for each gene. Forward and reverse sequences were assembled and edited using Geneious (version 9.1.8 Biomatters; http://www.geneious.com). MAFFT v7.017 [50] was used to align trimmed DNA sequences for each gene under E-INS-i algorithm for nuclear genes and G-INS-i for COI [51]. The three alignments were concatenated and manually edited for ambiguous regions using Geneious v1.9.8. In order to root the tree, three species of Misophrioida Gurney, 1933 and two species of Canuelloida Khodami, Vaun MacArthur, Blanco-Bercial & Martinez Arbizu, 2017 [52] were chosen as outgroups. The phylogenetic analyses were conducted using Bayesian inferences using MrBayes MPI version v3.2.2 × 64 [53, 54]. GTRGAMMA (General time reversible following gamma shape distribution) substitutional model were used for phylogenetic analyses as the best nucleotide fitting model for 18S and 28S rRNA calculated by jModeltest v0.1.1 [55] under Java. The codon model was implemented for the COI partition following the nexus block: lset Nucmodel = codon, code = metmt, omegavar = M3, nst = mix and rates = gamma. Two different MrBayes character partitions were provided and compared in two independent MrBayes jobs for the phylogenetic trees: job 1) considering 18S and 28S in one partition under nst = 6 (GTR) rate = gamma; job 2) allocating individual partition for 18S and 28S in which the nst = 6 and rate = gamma were applied to each partition separately. Posterior probabilities were estimated using 8,792,000 generations through four simultaneous Markov Chains Monte Carlo for the number of 2 runs and sample frequency of every 1000 trees. The average standard deviation of split frequencies between 2 runs reached the value of 0.007 for job 1 and 0.004 for job 2. The ESS (estimated sampling size) is more than 300 for all estimated parameters in job1 in which the ESS value of the LnL has been estimated to 1408–1561 for both runs. However in Job 2 the ESS value of some parameters are less than minimum accepted (Supplementary information) which indicates that the posterior was not effectively sampled during this single run. A 50% majority rule consensus tree with median branch lengths was made, discarding the 25% of the first trees (relburnin = yes) from each job individually. All generated trees (.t files) and estimated priors (.p files) are available as supplementary information for both MrBayes jobs (Additional files 2 and 3). The gene fragments sequenced in this study are available in Genbank following the accession numbers MN536817 - MN536902 for 18S rRNA, MN536171 - MN536215 for COI mtDNA and MN535552 - MN535623 for 28S rRNA.

**Table 2:** PCR and sequencing primers used for this study

Primer	Fragment	Annealing Temperature
18SE-F [44]	18S rRNA	51 °C
18SL-R [45]		
F1 [46]		
CF2 [46]		
CR1 [46]		
R2 [46]		
28S-F1a [47]	28S rRNA	51 °C
28S-R1a [47]		
LCO 1490 [48]	COI mt DNA	44 °C
HCO 2198 [48]		
Cop-COI-2189R [49]		

## Systematic part

Definition of the subfamily Cerviniellinae **subfam. nov.**, re-definition of the subfamilies Cerviniinae, Cerviniopseinae, Pontostratiotinae and Aegisthinae have been provided bellow. All abbreviation used in this section has been lifted in section 7 (List of abbreviations). Caudal setae labeled as follows: I- anterolateral accessory seta; II- anterolateral (lateral) caudal seta; III- posterolateral (outermost) caudal seta; IV-outer terminal (terminal median external) caudal seta; V-inner terminal (terminal median internal) caudal seta; VI-terminal accessory (innermost) caudal seta; VII- dorsal seta; nomenclature follows Huys and Boxshall [56]. The terms furca and telson are used following Schminke [57].

### Groundpattern of Aegisthidae (after [21])

*Female.* Prosome and urosome clearly separated, prosome consisting of cephalosome and 4 free pedigerous somites; first pedigerous somite separated from dorsal cephalic shield. Urosome 5-segmented comprising of fifth pedigerous somite, genital double-somite and three free abdominal somites. Anal somite elongate, tapering posteriorly. Furca more than twice as long as wide, bearing 7 furca setae. Antennule (A1) 8-segmented; armature formula: 1(1 s), 2(12 s), 3(14 s + 1ae), 4(2 s), 5(3 s), 6(2 s), 7(2 s), 8(6 s + 1aecrothek). Oligoarthra segments 3 + 4 fused. Antenna (A2) with coxa, incomplete allobasis and 1 free endopodal segment; basis and endopod 1 (enp1) fused, bearing 1 seta each; enp 2 with 1 spine and 2 setae on lateral margin and 7 distal setae; Exopod (exp) 4-segmented, armature formula: 1(2 s), 2(1 s), 3(1 s), 4(3 s). Mandible (Md) with coxa bearing a well-develop gnathobase; palp formed by basis, enp and exp.; basis with 4 setae; enp large (at least 2 times longer than wide), with 1 spine and 2 setae laterally and 7 distal setae, spine I lacking; exp. 4-segmented with 2,1,1,2 setae. (Maxillula) Mx1 comprising praecoxa, coxa, exp. and basis fused with enp; epipodite represented by 2 setae; fused basis and enp with 14 setae; exp. reduced in size bearing 3 setae. (Maxilla) Mx2 4-segmented comprising syncoxa, allobasis and three segmented enp; endopodal element 11 of allobasis developed as large, strong spine inserted on posterior surface. (Maxilliped) Mxp 4-segmented, comprising syncoxa, basis and 2-segmented enp; coxa with incorporated endites; basis with 1 spine and 1 seta; enp with 3 s, 2sp + 2 s. (Swimming legs 1 to 4) P1-P4 with 3-segmented rami. P5 without endopodal lobe; exp. more than twice as long as wide, armature: 1 inner spine, 3 outer spines, and 1 setae and 2 spines around apex. P6 with 3 setae.

*Male:* Sexual dimorphism in body size, genital segmentation, A1, P5 and P6. Caudal setae I and II transformed into strong spines. Urosome (Urs) 6-segmented, comprising of fifth pedigerous somite and 4 abdominal somites, 2 spermatophores. A1 10-segmented; armature: 1(1 s), 2(11 s + 1ae), 3(6 s + 1ae), 4(2 s), 5(3 s + 1ae), 6(2 s), 7(2 s), 8(3 s), 9(4 s), 10(10s + 1 aecrothek); fusion of Oligoarthra segments 2 and 3, 10 and 11, 12 to 14. P5 without endopodal lobe, basis separated from coxa, and 3-segmented exp. P6 with 3 setae.

### Cerviniellinae subfam. nov.

Type genus: *Cerviniella* Smirnov, 1946

Other genera; *Eucanuella* Scott, 1901, *Hase* Corgosinho, Kihara, Schizas, Ostmann, Martinez Arbizu & Ivanenko, 2018.

Body robust, dorso-ventrally compressed; first pedigerous somite incorporated into the cephalosome forming a carapace-like extension of the cephalosome in *Cerviniella* and *Eucanuella* (Fig. 3e) and completely separated in *Hase* (arrow in Fig. 3b). Rostrum small and fused to cephalic shield; **A1 short, 5–7 segmented with many setae transformed into strong spines** (arrow in Fig. 3f), A2 shortened, with complete or incomplete allobasis, exp. 4-segmented with setae formula 2.1.1.2 or 2.1.1.3 (Fig. 3f), **apical elements of enp 2 transformed into strong spines** (arrow in Fig. 3c). Maxillular epipodite absent or represented by one seta. **P1-P4 ramus bent inwards** (arrow in Fig. 3a, e); **P2-P4 enp reduced** (arrow in Fig. 3d)**,** with a maximum of 2 segments per ramus, in many cases reduced to a single segment or absent; exp. with 1, 2 or 3 segments. **Urosome with strong hook-like lateral projections** on genital double somite in *Cerviniella* species (arrow in Fig. 3e, d) and on last Urs in *Hase* (Fig. 3b). Furca divergent or parallel; not elongated, normally as long as anal somite or shorter; anterolateral accessory seta (I) inserted near to proximal margin, inserted at 10–30% of total length of furca (arrow in Fig. 3e); anterolateral caudal seta (II) inserted at 20–50% of furca (arrow in Fig. 3e).

### Cerviniinae Sars. M. 1903

Type genus: *Cervinia* Norman in Brady, 1878

Other genera: *Expansicervinia* Montagna, 1981; *Paracerviniella* Brodsky, 1963.

Body slightly elongated, with clear separation between prosome and urosome; **first pedigerous somite free** (arrow in Fig. 5a, b). Rostrum slightly produced and fused to cephalic shield; A1 approximately the same length as cephalosome, 6–8 segmented without spine-like seta, A2 with complete or incomplete allobasis, exp. 4-segmented with setae formula 2.1.1.3 in all species (Fig. 5c). Maxillular epipodite represented by one seta. P1-P4 rami in normal position; P1-P4 exp. 3-segmented, P1-P4 enp 2–3 segmented. **Female P5 1-segmented** (arrow in Fig. 5a). Urs somites without hook-like lateral projections. Furca divergent or parallel; not elongated, normally as long as or slightly longer than anal somite; anterolateral accessory seta (I) inserted near proximal margin (arrow in Fig. 5b), anterolateral caudal seta (II) inserted at posterior half of furca (arrow in Fig. 5b).

Remarks: the inclusion of the genus *Paracerviniella* within this family should be taken carefully, this taxon was described based only in a single male specimen. The variability and high dimorphism present in the Aegisthidae males makes difficult to decide the correct position of this taxon, the genus is temporally retained here until further specimens allowed a complete comparison.

### Cerviniopseinae Brotskaya, 1963

Type genus: *Cerviniopsis* Sars, G. O. 1903.

Other genera: Aegisthidae gen. 2; *Hemicervinia* Lang, 1935, *Herdmaniopsis* Brotskaya, 1963*; Siphonis* Mercado-Salas, Khodami & Martínez Arbizu, 2019; *Stratiopontotes* Soyer, 1970; *Tonpostratiotes* Itô, 1982.

Body elongated typically with first pedigerous somite free (arrow in Fig. 4d), when fused to cephalosome the suture is always conspicuous. Rostrum triangular in shape and fused with cephalosome, in some species well developed. Prosome without ornamentation of elaborate dorsal processes (Fig. 4a, d); A1 5–8 segmented, setae slender and long (arrow in Fig. 4c) and without spinous processes on first four segments, first and second segments not elongated. A2 with complete or incomplete allobasis, free endopodal segment about the same size of allobasis; exp. 4-segmented with setae formula 2.1.1.2 or 1.1.1.2 (Fig. 4e). **Basis of mandibular palp quadrated** as long as wide or slightly wider, enp and exp-1 not elongated (arrow in Fig. 4a). Maxillular epipodite represented by one seta (in some species absent). P1-P4 rami straight (not bended), usually with elongated exp. and enp (Fig. 4b, e); P1-P4 enp and exp. 3-segmented (members of *Hemicervinia* and Aegisthidae gen. 2 with P1 enp 2-segmented). **Furca adpressed (parallel)** along entire inner margin (arrow in Fig. 4b, e), usually about same size or slightly longer than total length of urosome; anterolateral accessory seta (I) inserted at 20–50% of total length of furca (arrow in Fig. 4a); anterolateral furcal seta (II) inserted at 80–90% of furca (arrow in Fig. 4a).

### Aegisthinae Giesbrecht, 1893

Type genus: *Aegisthus* Giesbrecht, 1891

Other genera: *Andromastax* Conroy-Dalton & Huys, 1999; *Jamstecia* Lee W. & Huys, 2000; *Nudivorax* Lee W. & Huys, 2000; *Scabrantenna* Lee W. & Huys, 2000.

Body elongated, with first pedigerous somite free. Rostrum fused to cephalosome (arrow in Fig. 6b), in some species not prominent but in other well developed (especially in *Aegisthus*). Prosome with or without ornamentation of elaborate processes (arrow in Fig. 6b); A1 6–7 segmented, with or without spinous processes (arrow in Fig. 6b); A2 with complete allobasis bearing only one small seta; **exp 2–3 segmented** (Fig. 6c). **Mandibular palp reduced to 1–2 segments bearing 2 setae** (arrow in Fig. 6c)**; maxillular epipodite and exopodite absent; Mxp reduced to 2–3 segments** (arrow in Fig. 6c)**; males with more reduced mouthparts.** P1 enp 2-segmented in all genera (suture between enp2 and enp3 usually conspicuous) with exception of *Aegisthus* where it retains 3-segmented state; P1-P4 exp. always 3-segmented, rami usually elongated. Furca adpressed (parallel) along entire margin (arrow Fig. 6a), slightly longer than total length of urosome in the genus *Nudivorax* or longer than the body in the other genera. **Anteroraleral accessory seta (I) reduced or absent**, when present always inserted at 20–30% of total length of furca; anterolateral caudal seta (II) always present and inserted at 50% of furca.

### Pontostratiotinae Scott, A. 1909

Type taxon: *Pontostratiotes* Brady, 1883

Body elongated, with first pedigerous somite free. **Rostrum triangular** and fused to cephalosome, not well developed. Usually with **elaborated dorsal process on the prosome** (arrow in Fig. 6e), some members without. A1 8-segmented, **spinous process on segments 1–2** always present (arrow in Fig. 6d, e) (some species with additional processes on segments 3–4); segments 1–3 elongated. A2 with complete or incomplete allobasis; free endopodal segment elongated, usually longer than allobasis; exp. 4-segmented with setae formula 2.1.1.2 or 0.1.1.2. **Basis of mandibular palp rectangular, always longer than wide; enp and exp-1 elongated, at least three times longer than wide and bent outwards, being almost parallel to basis.** Maxillular epipodite represented by one seta (in some species absent). Mx2 and Mxp following Aegisthidae ground pattern (arrow in Fig.6f). P1-P4 rami straight; enp and exp. elongated and 3-segmented. **Furca adpressed (parallel) along entire margin** (arrow in Fig. 6d); usually as long as or longer than total body length (in some species rami as long as urosome); anterolateral accessory seta (I) inserted at 20–30% of total length of furca (arrow Fig. 6d); anterolateral caudal seta (II) inserted at 60–90% of furca (arrow Fig. 6d).

## Supplementary information

**Additional file 1.** The second tree topology.

**Additional file 2.** MrBayes Job1.

**Additional file 3.** MrBayes Job2.

## Data Availability

The datasets generated and analyzed during the current study including sequences alignment, nexus blocks used for MrBayes phylogenetic analyses, generated trees and the MrBayes log files (information about the runs) are available as supplementary information file 1 and 2 and all sequences are available in GenBank following the accession numbers MN536817 - MN536902 for 18S rRNA, MN536171 - MN536215 for COI mtDNA and MN535552 - MN535623 for 28S rRNA.

## Abbreviations

18S rRNA: Small subunit 18S ribosomal RNA
28S rRNA: Large subunit 28S ribosomal RNA
A1: Antennule
A2: Antenna
COI mtDNA: Cytochrome c oxidase subunit I mitochondrial DNA
Enp: Endopod
Exp: Exopod
Md: Mandible
Mx1: Maxillula
Mx2: Maxilla
Mxp: Maxilliped
P1-P6: First to sixth thoracopod
Urs: Urosomite(s)
