# Supplementary figures and images for: Genus level molecular phylogeny of Aegisthidae Gisbrecht, 1893 (Copepoda: Harpacticoida) reveals morphological adaptations to deep-sea and plagic habitats
Source: BMC Evol Biol. 2020 Mar 14;20:36. doi: 10.1186/s12862-020-1594-x (PMC7071595; doi:10.1186/s12862-020-1594-x)

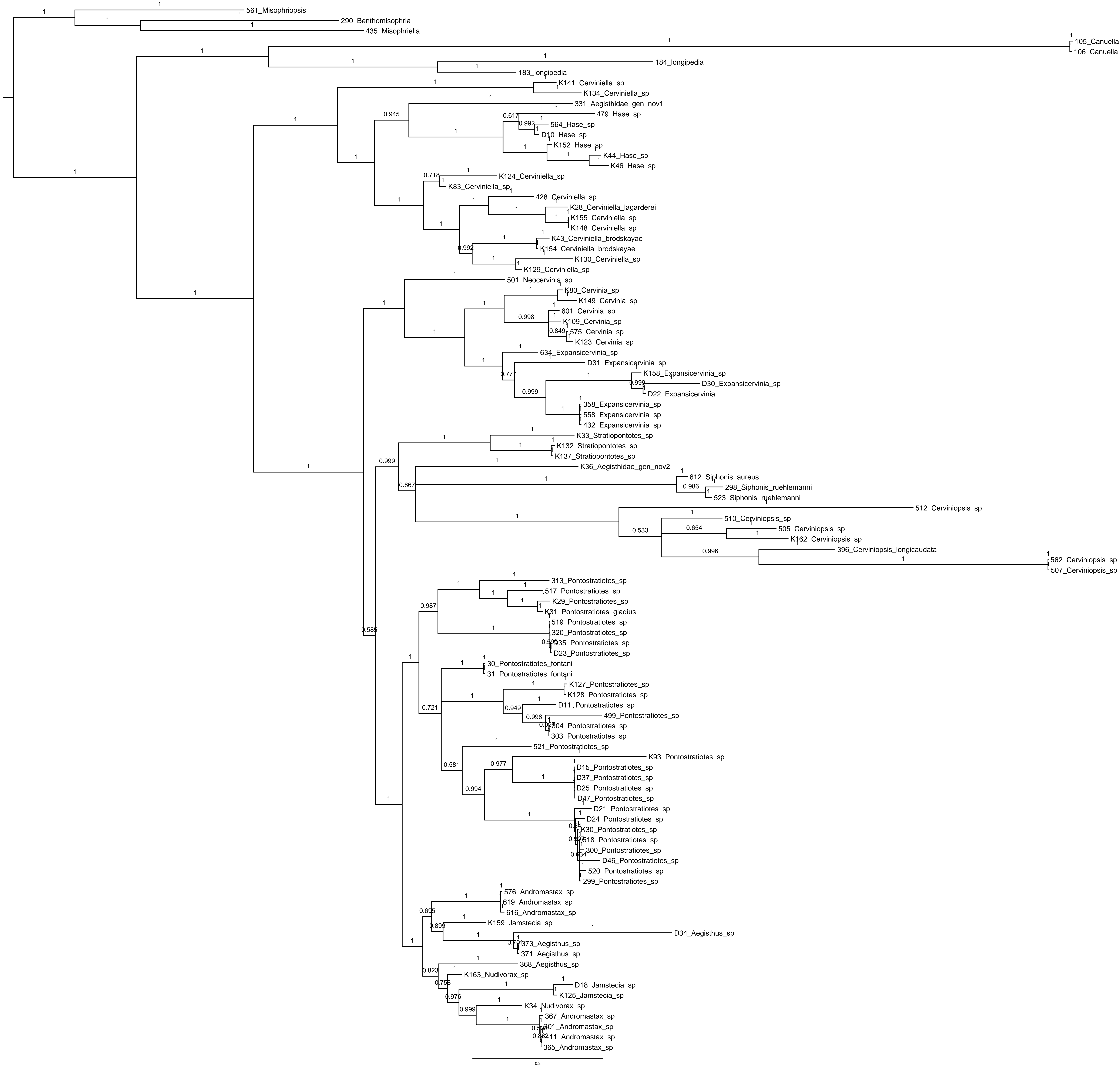

Supplement: Supplementary file 1 — Additional file 1. The second tree topology. [file 12862_2020_1594_MOESM1_ESM.pdf]

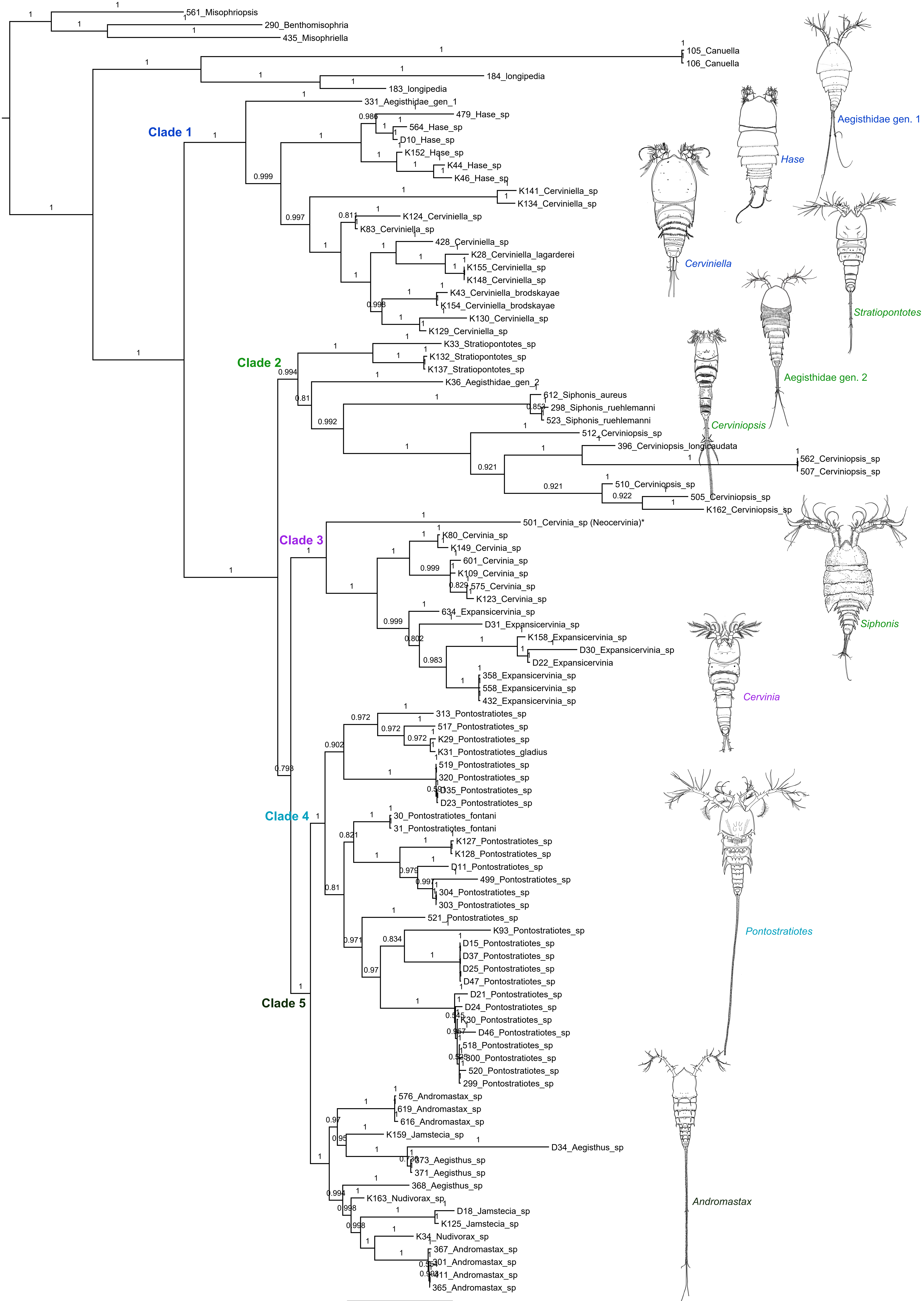

Supplement: Supplementary file 2 — Additional file 2. MrBayes Job1. [file 12862_2020_1594_MOESM2_ESM.zip › Job1_supplementary_info/18S_28S_COI_codon_renamed_modified.nex.con.tre.habitus.pdf]

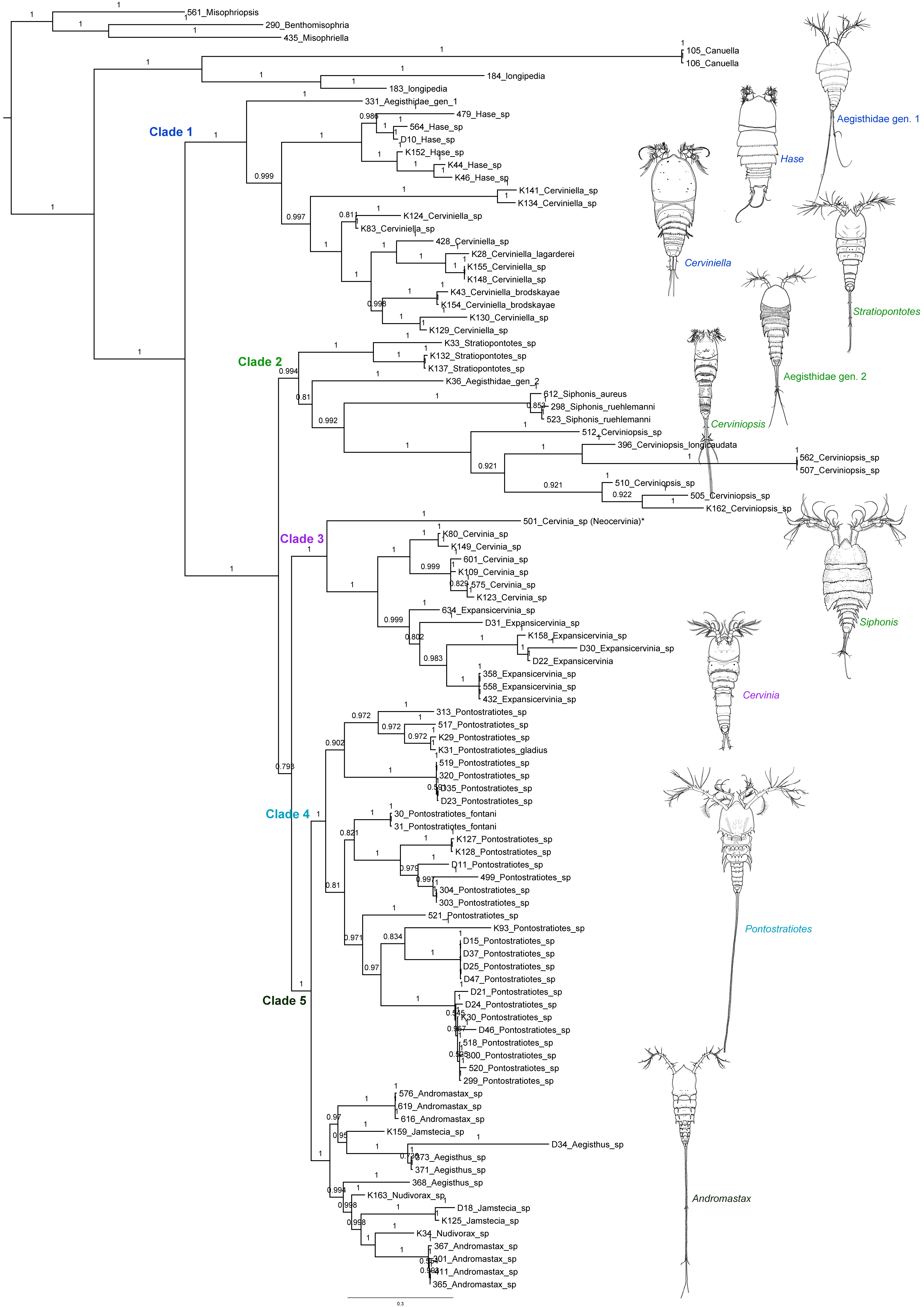

Supplement: Supplementary file 2 — Additional file 2. MrBayes Job1. [file 12862_2020_1594_MOESM2_ESM.zip › Job1_supplementary_info/18S_28S_COI_codon_renamed_modified.nex.con.tre.habitus.tif]

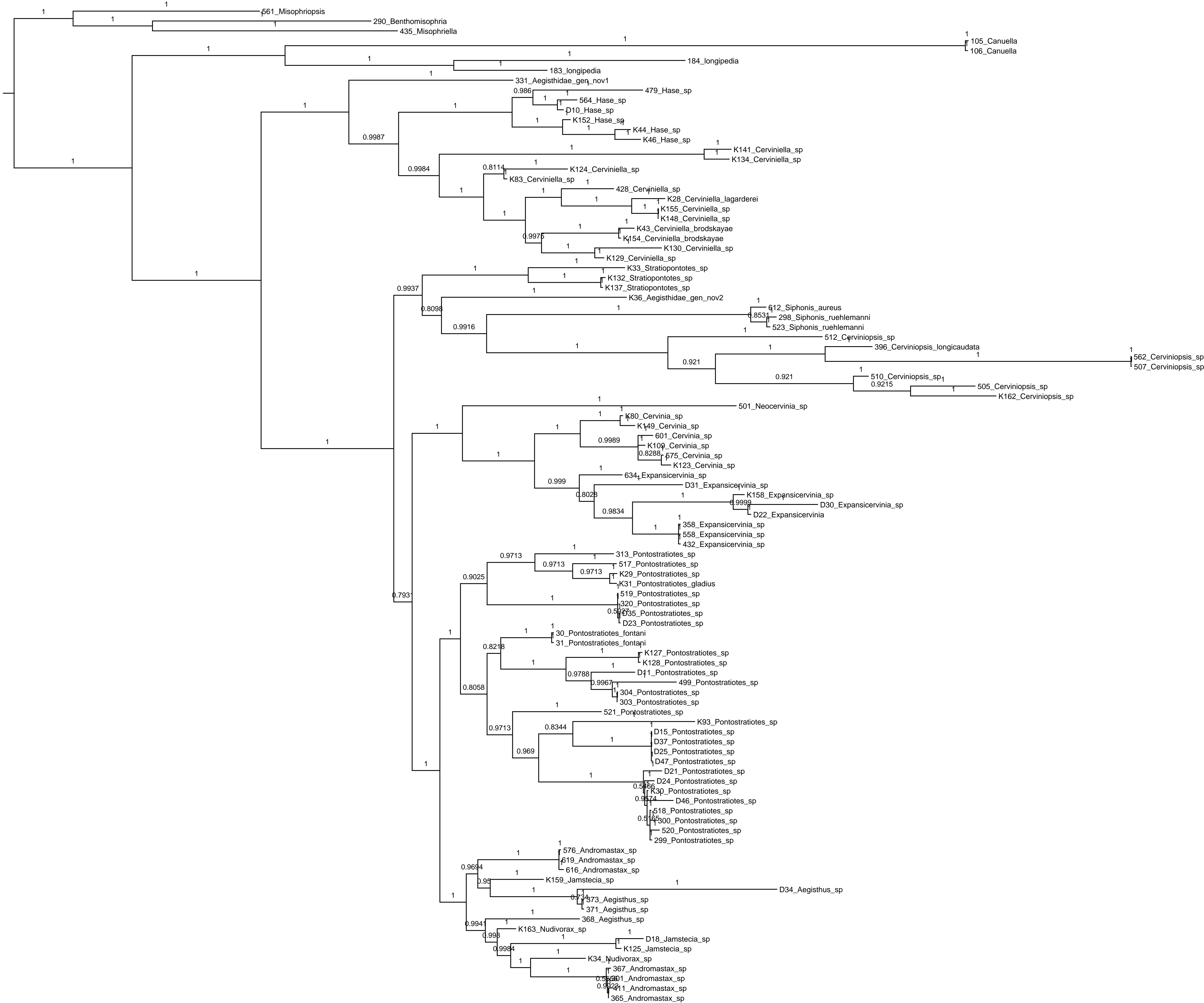

Supplement: Supplementary file 2 — Additional file 2. MrBayes Job1. [file 12862_2020_1594_MOESM2_ESM.zip › Job1_supplementary_info/18S_28S_COI_codon_renamed_modified.nex.con.tre.pdf]
